# The development and validation of tau PET tracers: current status and future directions

**DOI:** 10.1007/s40336-018-0290-y

**Published:** 2018-07-20

**Authors:** Nobuyuki Okamura, Ryuichi Harada, Aiko Ishiki, Akio Kikuchi, Tadaho Nakamura, Yukitsuka Kudo

**Affiliations:** 1 Division of Pharmacology, Faculty of Medicine, Tohoku Medical and Pharmaceutical University, Sendai, Japan; 2 Department of Pharmacology, Tohoku University School of Medicine, Sendai, Japan; 3 Institute of Development, Aging and Cancer, Tohoku University, Sendai, Japan; 4 Department of Neurology, Tohoku University School of Medicine, Sendai, Japan

**Keywords:** Tau, Positron emission tomography, Neurofibrillary tangles, Alzheimer’s disease, Neurodegeneration, Aging

## Abstract

**Purpose:**

To provide an overview on positron emission tomography (PET) imaging of tau pathology in Alzheimer’s disease (AD) and other neurodegenerative disorders.

**Results:**

Different classes of tau tracers such as flortaucipir, THK5317, and PBB3 have been developed and utilized in previous clinical studies. In AD, the topographical distribution of tracer binding follows the known distribution of neurofibrillary tangles and is closely associated with neurodegeneration as well as the clinical phenotype of dementia. Significant retention of tracers has also been observed in the frequent site of the 4-repeat (4R) tau isoform deposits in non-AD tauopathies, such as in progressive supranuclear palsy. However, in vitro binding studies indicate that most tau tracers are less sensitive to straight tau filaments, in contrast to their high binding affinity to paired helical filaments of tau (PHF-tau). The first-generation of tau tracers shows off-target binding in the basal ganglia, midbrain, thalamus, choroid plexus, and venous sinus. Off-target binding of THK5351 to monoamine oxidase B (MAO-B) has been observed in disease-associated brain regions linked to neurodegeneration and is associated with astrogliosis in areas of misfolded protein accumulation. The second generation of tau tracers, such as [^18^F]MK-6240, is highly selective to PHF-tau with little off-target binding and have enabled the reliable assessment of PHF-tau burden in aging and AD.

**Conclusions:**

Tau PET tracers have enabled in vivo quantification of PHF-tau burden in human brains. Tau PET can help in understanding the underlying cause of dementia symptoms, and in patient selection for clinical trials of anti-dementia therapies.

## Introduction

Misfolded protein accumulation is a common pathogenic mechanism in several neurodegenerative disorders, such as Alzheimer’s disease (AD) and Parkinson’s disease. The neuropathology of AD is characterized by the deposition of senile plaques (SPs) and neurofibrillary tangles (NFTs), which are misfolded aggregates of amyloid-β (Aβ) and hyperphosphorylated tau proteins, respectively [1]. Definitive diagnosis of AD can be made by postmortem autopsy confirmation of these protein lesions in the brain. Aβ is a 39–43 amino acid peptide derived from the proteolytic cleavage of the amyloid precursor protein (APP) by β-secretase (BACE1) and γ-secretase. This protein has been regarded as an initiator of the neurodegenerative process in AD [2]. Pathogenic mutations in the APP gene or presenilin genes cause the overproduction of Aβ and early-onset familial AD. In sporadic AD, impaired clearance of Aβ is considered to be the main cause for the accumulation of Aβ in the brain [3]. The amyloid cascade hypothesis has been widely accepted for explaining the etiology and pathogenesis of AD [4]. The deposition of Aβ is the initial pathological event in AD leading to the formation of SPs and then to NFTs, neuronal loss, and ultimately cognitive decline. Amyloid PET studies have demonstrated that neocortical accumulation of Aβ precedes neurodegenerative changes and cognitive decline in AD [5].

Tau protein, another key player in the pathogenesis of AD, is a microtubule-associated protein (MAP), which is abundantly expressed in the central nervous system (CNS) [6]. The accumulation of hyperphosphorylated tau protein in neurons is considered to be crucial in the neurodegenerative process of AD. Abnormal hyperphosphorylation of tau causes protein self-aggregation and the formation of paired helical filaments (PHF). Abnormal tau aggregation occurs not only in the AD brain, but also in a family of protein-misfolding diseases called tauopathies, which include frontotemporal dementia with parkinsonism linked to chromosome 17 (FTDP-17), Pick’s disease, progressive supranuclear palsy (PSP), corticobasal degeneration (CBD), and chronic traumatic encephalopathy [7]. There are six molecular isoforms of tau generated by alternative pre-mRNA splicing of a single gene transcript and classified according to their tubulin-binding domains as 3-repeat (3R) and 4-repeat (4R) tau proteins. Equal amounts of 3R and 4R tau isoforms exist in the normal brain. In PHF-tau, there is also an equal balance of 3R and 4R isoforms. The changes in the 3R and 4R tau ratio can cause abnormal tau accumulation and lead to neurodegeneration as in tauopathies [8]. For example, the 3R tau isoform is predominantly accumulated in Pick’s disease, whereas the 4R tau is more abundant in PSP, CBD, and argyrophilic grain disease [8]. In 4R tauopathies, tau aggregates are also observed in glial cells and form disease-specific astroglial lesions, such as tufted astrocytes in PSP and astrocytic plaques in CBD. Frontotemporal lobar degeneration linked to microtubule-associated protein tau (MAPT) mutations involves 3R tau or 4R tau, or a combination of both isoforms. The spatial distribution of tau deposits is different for each tauopathy and is strongly associated with the clinical phenotype of these diseases.

## Characteristics of tau PET images

Table 1 summarizes the comparison between amyloid and tau PET images in AD. In contrast to diffuse and widespread distribution of amyloid tracers in the neocortex, retention of tau tracers is mainly observed in the inferior temporal and parietal cortices of AD patients. Neocortical PHF-tau formation is considered to be a downstream event after Aβ deposition in AD [9, 10]. Therefore, tau protein deposits are not frequent in the neocortex, even if widespread amyloid-β deposits exist during asymptomatic stages of AD [2]. Normal aging is also associated with accumulation of PHF-tau in the medial temporal lobe. The formation of NFTs in the absence of Aβ deposition is neuropathologically classified as primary age-related tauopathy (PART) [11]. Significant tau tracer retention in the temporal lobe has been reported even in cognitively normal older people [12]. Amyloid PET studies have shown little association between amyloid burden and the clinical severity of dementia [13]. In contrast, tau PET studies have shown a close relationship between tau pathology and the clinical severity of dementia [5]. During aging and in the very early stages of AD, tau tracer retention is usually confined to the medial temporal cortex. After dementia symptoms appear, neocortical tau pathology progresses rapidly following the onset of dementia and adhering to a stereotypical spatial pattern as the clinical symptoms progress [1]. Thus, tau imaging may allow the accurate staging of AD [14] and could prove useful in predicting the prognosis of dementia. Recent PET studies have shown that the topographical distribution of tau tracer retention overlaps with that of other neurodegenerative markers such as fluorodeoxyglucose (FDG)-PET and structural magnetic resonance imaging (MRI). PET imaging enables the longitudinal assessment of tau pathology progression and the understanding of the natural history of tau pathology progression.

**Table 1:** Comparison between amyloid and tau PET in Alzheimer’s disease (AD)

	Amyloid PET	Tau PET
Tracers	[^11^C]PiB [^18^F]Flutemetamol [^18^F]Florbetapir [^18^F]Florbetaben [^18^F]NAV4694	[^18^F]Flortaucipir (AV1451), [^18^F]T808, [^18^F]THK5117(5317), [^18^F]THK5351, [^11^C]PBB3, [^18^F]PM-PBB3, [^18^F]RO69558948, [^18^F]GTP1, [^18^F]MK6240, [^18^F]PI2620
Binding target	Amyloid-β fibrils (in senile plaques)	PHF-tau (in neurofibrillary tangles, neuropil threads, dystrophic neurites)
Frequent site of on-target binding	Neocortex (diffuse)	Temporo-parietal cortex (in amnestic AD)
Diversity of topographical tracer distribution	Low	High (associated with clinical phenotype of AD)
Association with clinical severity of dementia	Low	High (in tracer density and spatial extension)
Association with neurodegenerative markers (FDG-PET, gray matter loss)	Low	High
Clinical applications	1. Patient selection and evaluation of the therapeutic effect in clinical trials for anti-Aβ drugs 2. Differentiation between Aβ-positive and Aβ-negative conditions 3. Preclinical assessment of AD-related pathology for early intervention	1. Patient selection and evaluation of the therapeutic effect in clinical trials for anti-Aβ and anti-tau drugs 2. Accurate disease staging 3. Assessment of disease progression 4. Prediction of prognosis 5. Differential diagnosis of tauopathies

The development of disease-modifying therapies (DMTs) is ongoing as an initiative to decrease the number of patients with AD. Considering that the accumulation of Aβ starts before the clinical onset of AD, it is ideal to begin using DMTs before the clinical symptoms appear. Amyloid PET has been widely used for patient selection and the evaluation of treatment efficacy in clinical trials of anti-amyloid drugs. However, Aβ-targeted therapies have been unsuccessful to date. Tau plays a key part in the formation of NFTs and might, therefore, represent an important therapeutic target in AD [5, 15]. Based on previous studies, anti-tau immunotherapies and tau aggregation inhibitors appear to be promising therapeutic strategies in AD [16]. Tau PET has the potential to facilitate the monitoring of treatment efficacy of new drugs and the identification of suitable patients who could enroll in the clinical trials evaluating these drugs. Progressive tau accumulation has also been observed in many neurodegenerative diseases and chronic traumatic encephalopathies [16]. Recent PET studies have shown the potential utility of tau imaging for the differential diagnosis of non-AD tauopathies [5]. Although it is uncertain whether currently available tau tracers can be used as biomarkers of non-AD tau deposits, early and accurate diagnosis of these diseases will facilitate preventive clinical trials in a broad range of tauopathies.

## First generation of tau tracers

**Fig. 1:**
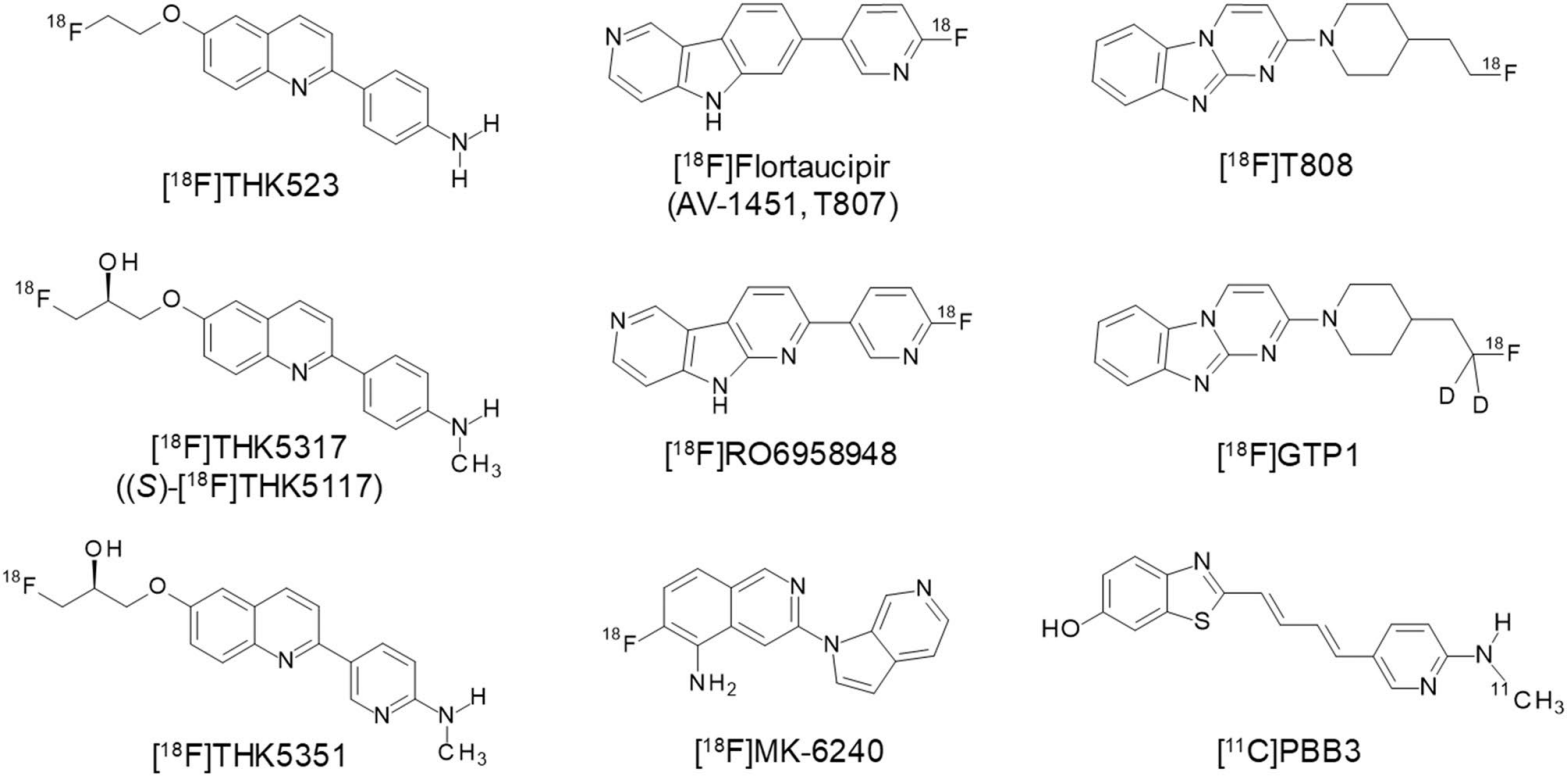
Chemical structures of tau PET tracers

Tau tracers are expected to bind tau protein fibrils at low nanomolar concentrations and should be highly selective for tau over Aβ, given that the concentration of Aβ is substantially higher than that of tau in the AD neocortex [17]. In vitro autoradiography of human brain sections has been used for the assessment of binding selectivity of radiotracers at low nanomolar concentrations [18]. High blood–brain barrier (BBB) permeability is also essential for brain PET tracers. Therefore, low molecular weight compounds have been used as tau PET tracers [18, 19]. Radiotracers should show sufficient amount of brain uptake after administration and be cleared rapidly from normal brain tissue. For quantitative analysis of PET images, it is also ideal that the radiolabeled metabolites do not enter the brain and/or bind to specific targets. The ^18^F-labeled tracer may be more suitable for high-throughput screening of tau burden than the ^11^C-labeled tracer. As shown in Fig. 1, several different classes of tau tracers have been developed and utilized in human studies. In contrast with the development of receptor ligands, there were no pre-existing small molecules that selectively bind to PHF-tau with high affinity. Furthermore, there are multiple binding sites on PHF-tau. This makes it difficult to conduct a blocking study using gold-standard ligand and limits the proof that the proposed radiotracers bind specifically to tau pathology in the human brain. Therefore, the binding specificity to tau protein deposits must be carefully validated in the development of new tau tracers.

### Quinoline derivatives

**Fig. 2:**
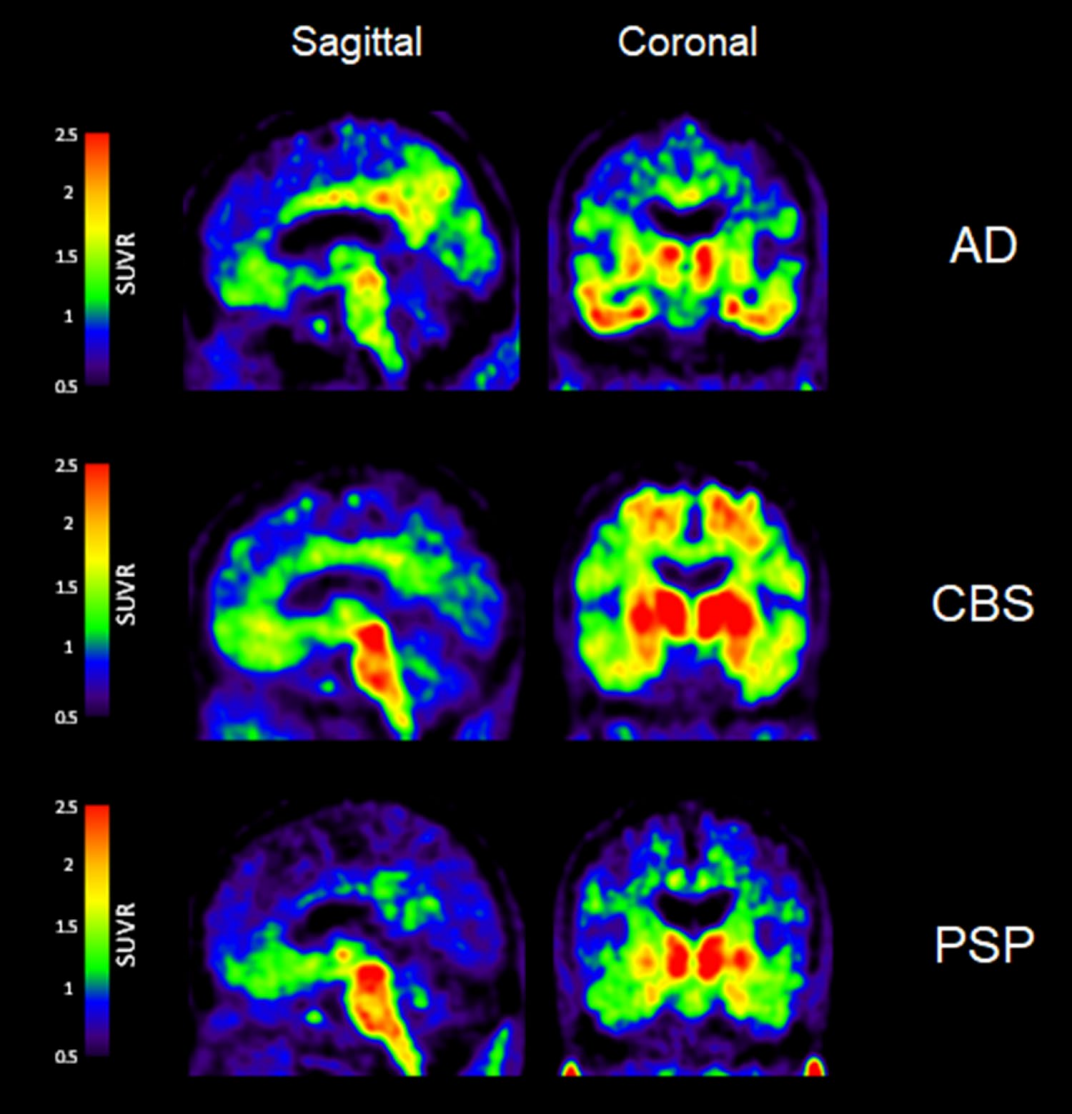
[^18^F]THK5351 PET images in patients with Alzheimer’s disease (AD), corticobasal syndrome (CBS), and progressive supranuclear palsy (PSP). In AD patients, THK5351 retention was high in the basal ganglia and the thalamus, reflecting the off-target binding to monoamine oxidase B (MAO-B). CBS patients showed prominent tracer retention in the basal ganglia, the precentral gyrus, and the midbrain. THK5351 retention in the midbrain was also elevated in PSP

**Fig. 3:**
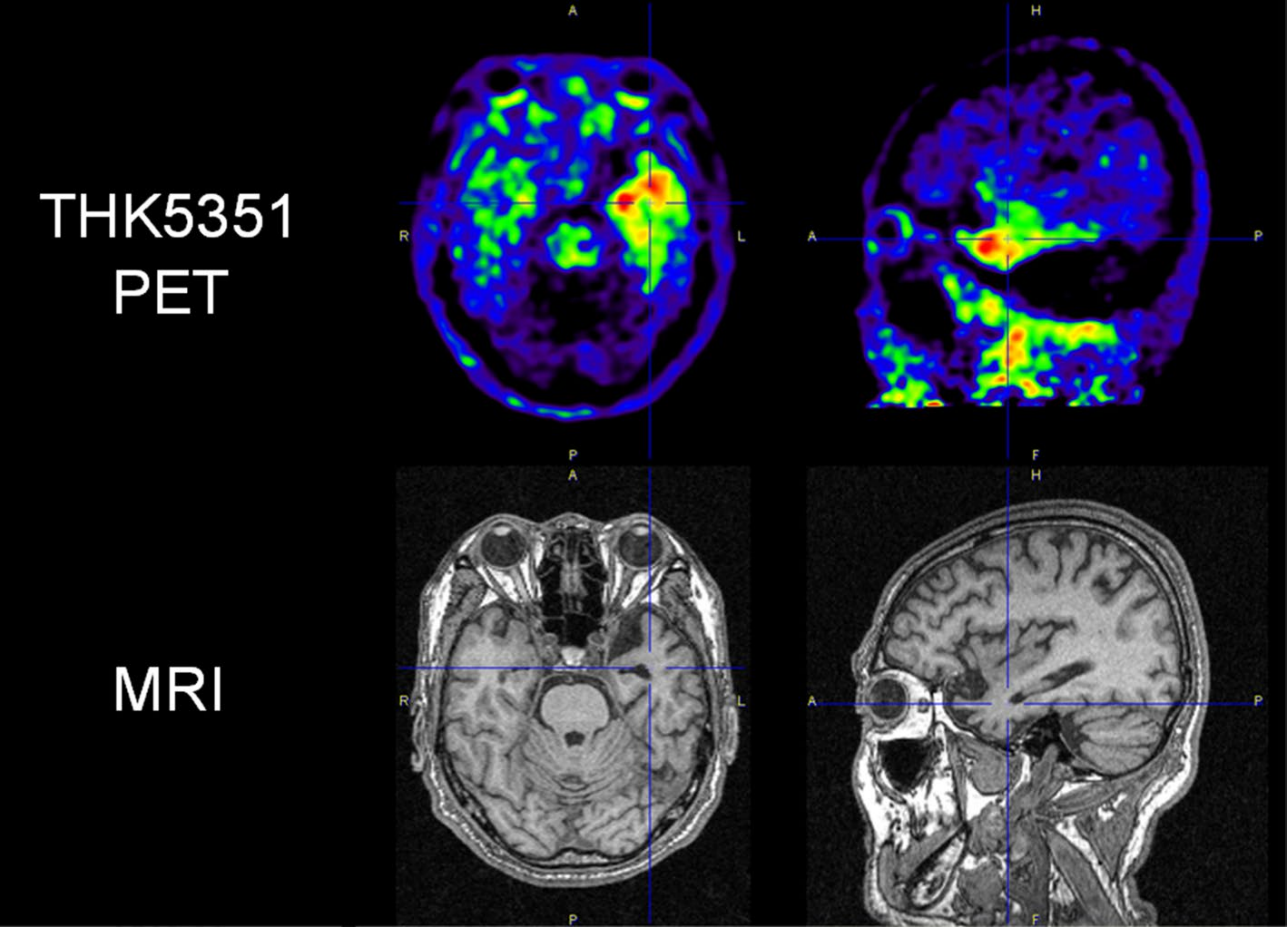
[^18^F]THK5351 PET images in patient with semantic variant primary progressive aphasia. Significant tracer retention was observed in the left anterior temporal pole. Figure courtesy of Dr. Ryota Kobayashi, Yamagata University

Quinoline derivatives, BF-158, BF-170, and BF-242 (THK-523), were first identified as potential candidates for tau tracers through the screening of β-sheet-binding compounds [20]. Notably, the [^18^F]THK523 showed higher affinity for tau fibrils than for Aβ fibrils [21]. Autoradiographic studies have demonstrated the accumulation of this tracer in AD brain regions containing a high density of PHF-tau. Small animal PET studies have also demonstrated significant tracer retention in brains of tau transgenic mice [22]. However, clinical PET studies have failed to visualize tau deposits in the gray matter of AD patients’ brains [23], possibly due to the insufficient binding affinity of this tracer to native tau conformation. [^18^F]THK5105 and [^18^F]THK5117 were additionally developed to improve the binding affinity to PHF-tau. These tracers exhibited binding affinity and selectivity to PHF-tau over Aβ in AD brain tissues [24]. In proof-of-concept PET studies, these radiotracers clearly differentiated the brains of patients with AD from those of healthy controls [25–27]. Furthermore, the amount of tracer retention in the neocortex was significantly correlated with the clinical severity of dementia [25]. Longitudinal PET studies have also demonstrated a significant correlation in changes of tracer binding with cognitive decline in AD patients [28]. The following clinical studies have been performed using the (*S*)-enantiomer of THK5117, because the (*S*)-enantiomer of quinoline derivatives showed better pharmacokinetics than the (*R*)-enantiomer [29, 30]. High [^18^F]THK5317 ((*S*)-[^18^F]THK5117) retention has been observed in cases of mild cognitive impairment (MCI) and AD dementia [31] and has been found to be negatively associated with cognitive performance [32]. One of the drawbacks of THK5117 and THK5317 is a non-negligible white matter binding, which is possibly due to the binding of the radiotracers to the β-sheet structures of the myelin basic protein. THK5351 was then developed to reduce its binding to the white matter tissue [33]. In human study, THK5351 exhibited faster white matter clearance and higher specific binding to AD tau-associated regions of interest (ROI) than THK5317 [34]. The spatial pattern of THK5351 binding in the neocortex was similar to that of THK5317 and was also found to be associated with the clinical phenotype of dementia [35]. Moreover, THK5351 retention was found to be correlated with extra-hippocampal subregional atrophy rather than hippocampal subfields, suggesting different underlying mechanisms of atrophy in early AD [36]. As shown in Fig. 2, significant THK5351 retention was also observed in the midbrain and the globus pallidus of PSP patients [37–39], which was found to be highly correlated to the clinical severity of PSP [39]. In patients with corticobasal syndrome (CBS), asymmetric THK5351 retention was observed in the frontal, parietal, and globus pallidus, contralaterally to the side associated with greater cortical dysfunction and parkinsonism [40]. In recent studies, doubt was cast on the selectivity of THK5351 to tau, because the tracer uptake was high in the basal ganglia, the thalamus, and the brainstem [41]. Furthermore, significant tracer retention has been observed in the brain of patients with semantic variant primary progressive aphasia (Fig. 3), which is infrequently associated with tau protein accumulation [42, 43]. These findings may be explained by off-target binding of THK5351, which will be discussed below.

### PBB3

PBB3 was designed to detect a broad range of tau pathologies in the human brain [44]. In an autoradiography of the hippocampus of AD patients, specific tracer binding was observed in the CA1 and subiculum areas, where high density of fibrillar tau aggregates exist [44]. In an in vitro binding analysis, PBB3 were found to bind preferentially to dystrophic neurites as well as NFTs in AD brains than flortaucipir [45]. In clinical PET studies, the binding of [^11^C]PBB3 was detected in the medial temporal region of AD patients. Tracer retention in the medial temporal region, the precuneus, and the frontal cortex was well correlated with cognitive decline. [^11^C]PBB3 binding was also associated with gray matter atrophy [46]. As is the case with THK5351, [^11^C]PBB3 retention was also observed in the putamen, the midbrain, the globus pallidus, and the substantia nigra of patients with PSP [47]. Laterality of tracer signals in the basal ganglia was also observed in patient with CBS [44], suggesting the binding of this tracer to 4R tau lesions. Recent PET studies have suggested different binding targets between PBB3 and THK5351 [48] with no significant correlation in the load and regional distribution between PBB3 and THK5351. There are several technical issues on the use of [^11^C]PBB3, including lower dynamic range, metabolic instability, and off-target binding in the basal ganglia, the longitudinal sinus and the choroid plexus [5]. The ^18^F-labeled derivatives ([^18^F]AM-PBB3 and [^18^F]PM-PBB3) have been developed and clinically tested to improve these drawbacks of [^11^C]PBB3.

### Flortaucipir ([^18^F]AV-1451)

**Fig. 4:**
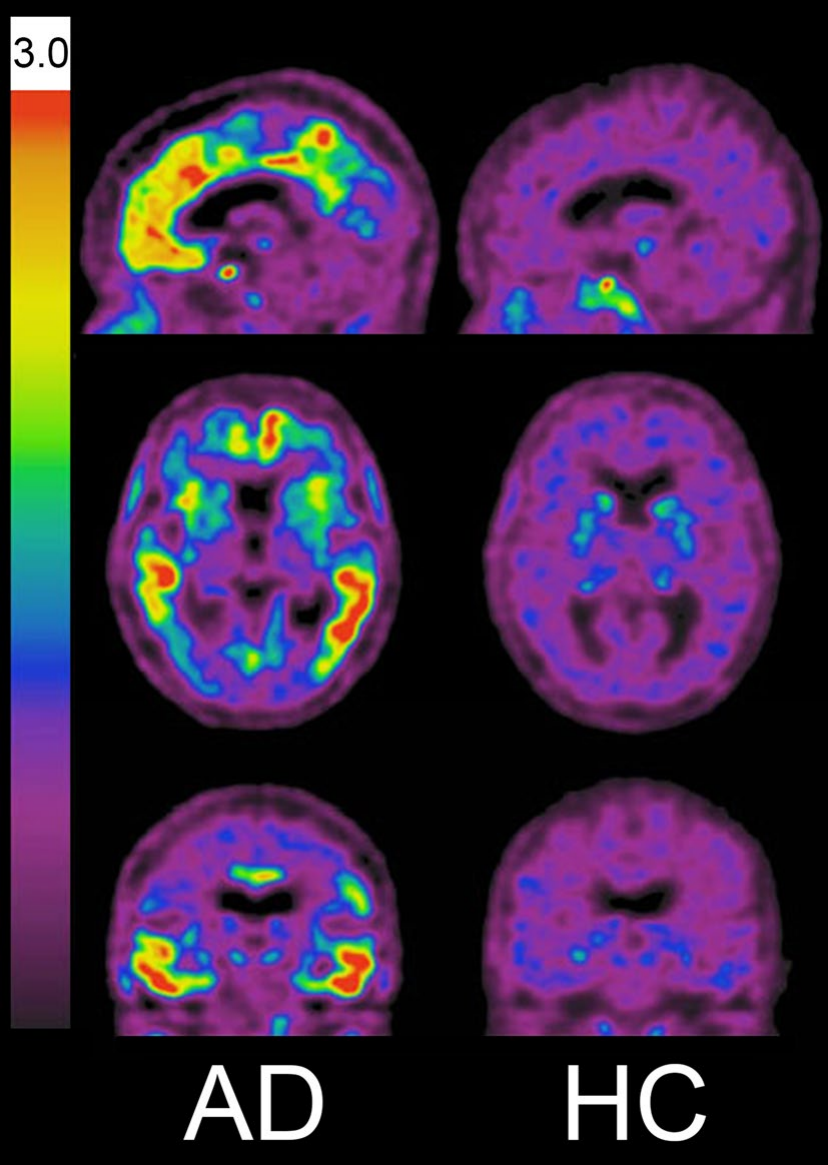
[^18^F]Flortaucipir PET images in patient with Alzheimer’s disease (AD) and healthy control (HC). Tracer retention was markedly elevated in the neocortex of AD patient Figure courtesy of Dr. Victor Villemagne, University of Melbourne

Benzimidazole pyrimidine derivatives were identified as candidates for tau PET tracers. [^18^F] flortaucipir, also known as [^18^F]AV-1451 (T807), and [^18^F]T808 exhibit high binding affinities to the PHF-tau, and high binding selectivity for tau over Aβ in AD brains [49–51]. Autoradiography studies have demonstrated that flortaucipir binding colocalizes with tau deposits, but not with Aβ, α-synuclein, or TDP-43 protein deposits [49, 52]. Additionally, postmortem analysis revealed that flortaucipir binding correlates with postmortem NFT Braak staging in AD brains [53]. Clinical PET studies have shown preferential tracer retention in the inferior temporal and the posterior parietal cortices of AD patients (Fig. 4) [12, 54]. In amnestic AD cases, the spatial patterns of tracer distribution follow the known distribution of NFTs in AD [55]. Furthermore, the amount and area of tracer retention significantly correlate with the clinical severity of dementia. Compelling test–retest reproducibility for flortaucipir was observed across the neocortical and the mesial temporal lobe structures in healthy controls and AD patients [56].

The relationship between flortaucipir binding and cognition was observed even in cognitively sound older people [12]. Interestingly, neocortical flortaucipir retention was found in some of the preclinical AD cases, but was rarely found in amyloid-negative cases [57, 58]. These observations suggest that the neocortical Aβ deposition may trigger the spread of tau pathology from the medial temporal area to the broad neocortical areas. Age-related increases in flortaucipir retention was observed in the medial temporal lobe of amyloid-negative, normal subjects, suggesting the existence of age-related tau deposition in this population, which is neuropathologically classified as PART [11]. However, whether flortaucipir retention in this area truly reflects age-related tau pathology in cognitively normal subjects is yet to be confirmed.

The diversity of flortaucipir distribution has been observed in amyloid-positive MCI and AD [58, 59], which is associated with the age of onset. Early-onset AD cases exhibited greater flortaucipir uptake than late-onset AD cases in the prefrontal and the premotor, as well as in the inferior parietal cortex [60]. There is also a diversity of flortaucipir distribution in the neocortex of atypical AD cases [61, 62]. Patients with logopenic variant primary progressive aphasia demonstrated asymmetric, left greater than right, hemisphere tracer retention. In a case with posterior cortical atrophy, preferential flortaucipir retention was observed in the posterior cortical regions, which showed a compelling association with glucose hypometabolism and the clinical phenotype observed [63]. Notably, flortaucipir retention was correlated with regional gray matter atrophy in this population [62]. Increased neocortical flortaucipir retention has also been observed in some cases of dementia with Lewy bodies (DLB) [64, 65]. However, the amount of flortaucipir binding in DLB patients was relatively lower than that of AD patients [65]. Intriguingly, 4 out of 17 of the Lewy body disease cases with low amyloid burden had elevated flortaucipir binding in the inferior temporal cortex, suggesting that tau pathology in DLB may extend to the neocortex without amyloid burden [64]. Many researchers have examined the relationship between flortaucipir PET and cerebrospinal fluid (CSF) biomarkers. To this end, flortaucipir binding in the para-hippocampal gyrus and neocortex was shown to correlate with CSF-tau levels [66, 67]. However, flortaucipir retention was more strongly correlated with neurodegeneration and cognitive decline than with CSF-tau levels. Cross-sectional analysis suggested that these two biomarkers showed different dynamics at different stages of AD [68].

Furthermore, clinical studies of flortaucipir have been extended to a broad range of tauopathies. In PSP patients, significant flortaucipir retention was observed in the globus pallidus, the putamen, the subthalamic nucleus, and in the midbrain of PSP patients [69–73]. The globus pallidus is one of the brain regions where PSP patients can be distinguished from control and PD patients’ brains. The spatial pattern of tracer binding may be associated with the clinical phenotype of PSP although the clinical severity of PSP did not significantly correlate with flortaucipir binding in any region [72]. These clinical findings suggest a binding ability of flortaucipir to the 4R tau lesion in PSP. However, postmortem analysis of PSP cases failed to show detectable flortaucipir binding to 4R tau deposits and also showed a lack of significant correlation between in vivo flortaucipir retention and the accumulation of straight tau filaments [73]. Several studies have shown a poor association of flortaucipir binding with non-AD tauopathies that have preferential accumulation of either 3R or 4R tau [52, 73, 74] although some studies have shown moderate tracer binding in brain tissues from patients with Pick’s disease and FTDP-17 [75]. Technical problems may underlie the discrepancies between the in vivo PET and the in vitro autoradiography results. In the autoradiogram of brain tissues, alcohol is frequently used for washing the slides after incubation. The treatment with alcohol could remove the specific tracer binding on tau deposits and cause the underestimation of specific binding in the autoradiogram. Significant PET findings have also been observed in patients with CBS. CBS patients exhibited distinct patterns of flortaucipir retention in the motor cortex, the corticospinal tract, and the basal ganglia contralateral to the affected body side [76, 77]. In an autopsy-confirmed case with corticobasal degeneration (CBD), regional flortaucipir binding showed an excellent correlation with 4R tau burden, suggesting higher sensitivity of flortaucipir to 4R tau deposits in CBD, compared with other non-AD tauopathies [78]. In another autopsy-confirmed CBD case, flortaucipir retention was highly related to tau-positive threads, when compared to tangle pathology in CBD [79].

## Current challenges in tau imaging

Recent studies have exhibited high radiotracer retention in the area where tau pathology is not frequently observed in AD and other tauopathies (Table 2). In flortaucipir PET studies, off-target binding has been observed in the midbrain, the meninges, the choroid plexus, and the striatum. In the analysis of autopsy brain samples, flortaucipir was found to bind to vessels, iron-associated regions, the substantia nigra, calcifications in the choroid plexus, and to leptomeningeal melanin [74]. High radiotracer signals in the substantia nigra and in the meninges are considered to be associated with neuromelanin and melanocyte binding. Therefore, this tracer could be also used to measure the density of pigmented dopaminergic neurons in the substantia nigra [80]. Significant reduction of flortaucipir binding was demonstrated in the substantia nigra of PD patients [81]. Off-target binding in extra-brain regions has been also observed in tau PET tracers. High tracer signal in the eye might be associated with tracer binding to melanin in retinal pigment epithelial cell [52]. Furthermore, high density of flortaucipir binding was observed in brain hemorrhage lesions, which may reflect the binding of this tracer to hemosiderin. Another frequent site of off-target binding is the choroid plexus which is closely located to the hippocampus. It should then be noted that high tracer signals in the choroid plexus can spill over into the hippocampus and cause a misinterpretation of tau PET results. Nonetheless, it is still not very well understood why the tracers accumulate in the choroid plexus. The choroid plexus produces the CSF and is known as the major route of blood–cerebrospinal fluid barrier exchange [82]. It may be possible that radiotracers and/or their metabolites are trapped in the epithelial cells of the choroid plexus during the transport from the blood into the CSF. Another possibility is that tau deposits in epithelial cells contribute to PET signals in the choroid plexus [83]. Striatum is also a frequent site of off-target binding of tau tracers. High amount of [^18^F]flortaucipir, [^18^F]THK5351, and [^11^C]PBB3 binding has been observed in the basal ganglia of aged normal subjects. The amount of these tracers was significantly elevated in patients with PSP and CBS. However, there is a debate as to whether the tracer retentions reflect the specific binding of PET tracers to tau deposits or not. Age-related increases of flortaucipir binding have been observed in the basal ganglia of healthy controls. This binding may be associated with the accumulation of iron in this area [74, 84]. There is also a possibility that neuroinflammatory changes or other concomitant proteinopathies are associated with significant tracer retention in PSP-related brain regions [59]. Monoamine oxidase (MAO) is one of identified off-targets of tau PET tracers. MAO is present in the outer mitochondrial membrane and is involved in the degradation of monoamines such as dopamine and serotonin. This enzyme is classified into two isoforms, MAO-A and MAO-B. The concentration of MAO-B in the brain is ten times higher than that of MAO-A. Flortaucipir is reported to show high binding affinity to MAO-A [85]. Another study also showed high affinity binding of flortaucipir to MAO-B in vitro [86]. However, MAO-B blocking studies in humans demonstrated that flortaucipir did not significantly bind to MAO-B in vivo [87, 88]. Therefore, it is unlikely that MAO-B is the off-target substrate of flortaucipir in the basal ganglia. In clinical studies of patients with semantic variant of primary progressive aphasia, flortaucipir binding was detected in regions that are likely to contain TDP-43 [89]. Considering low binding affinity of flortaucipir to TDP-43 in vitro [52], significant flortaucipir signals may be caused by unidentified molecules that are associated with non-specific neurodegenerative processes. THK5351 showed greater off-target binding in the midbrain, the thalamus, and the basal ganglia with greater cortical uptake in FTD than flortaucipir [5, 90]. Off-target binding of THK5351 can be attributed to MAO-B [41, 91]. Our initial investigation using fixed brain tissues failed to detect the binding of this tracer to MAO-B in the basal ganglia [33], possibly due to the fact that fixation of brain tissues with formalin and paraformaldehyde can denature the native structures of enzymes. In vitro autoradiography of [^18^F]THK5351 using fresh-frozen sections demonstrated a reduction of [^18^F]THK-5351 binding after treatment with MAO-B inhibitors [41, 91]. Furthermore, prominent reduction of PET signal has been observed in the brain of patients with AD and PSP, after single oral administration of 10 mg selegiline, an irreversible MAO-B inhibitor [92]. For this reason, THK5351 should not be used as a biomarker of tau. Compared with THK5351, THK5117 and THK5317 showed lower binding affinity to MAO-B in a competitive binding assay using ^3^H-Deprenyl [93]. High density of focal THK5351 binding has also been reported in patients with semantic variant primary progressive aphasia [45] and cerebral infarction [94], suggesting the binding of this tracer to MAO-B-positive astrogliosis [41, 91]. There are several molecular targets for in vivo imaging of neuroinflammation. For instance, many translocator protein (TSPO) tracers have been developed and used for the imaging of activated microglia. Considering the high dynamic range of THK5351 signals in various neurodegenerative diseases, MAO-B would be a promising target for the imaging of neuroinflammation in the brain.

**Table 2:** Common off-target sites of the first-generation of tau PET tracers

Brain regions	PET tracers	Binding target
Basal ganglia	THK5351 Flortaucipir PBB3	Monoamine oxidase B (MAO-B) Monoamine oxidase A, minerals Unidentified
Midbrain	THK5351 Flortaucipir	MAO-B, neuromelanin Neuromelanin
Thalamus	THK5351	MAO-B
Hippocampus	THK5351	MAO-B
Choroid plexus	Flortaucipir, PBB3	Unidentified
Venous sinus	PBB3	Unidentified

## Second-generation tau tracers

After the development of the first-generation tracers, several pharmaceutical companies have been trying to improve the binding selectivity and pharmacokinetics of tau PET tracers. [^18^F]RO6958948 (RO-948), [^11^C]RO6931643 (RO-643), and [^11^C]RO6924963 (RO-963) were identified as high-affinity competitors at the ^3^H-T808 binding site on native tau aggregates in AD brain tissue [95]. These tracers showed high affinity for NFTs and excellent selectivity against Aβ plaques in AD brain tissues. Among them, [^18^F]RO-948 showed appropriate pharmacokinetic and metabolic properties in mice and non-human primates [95, 96]. Preclinical binding analysis also has suggested lower binding affinity of these compounds to MAO-A and MAO-B than the binding affinity of flortaucipir and THK5351. Importantly, the results from a first-in-human PET study were consistent with preclinical data [97]. [^18^F]RO-948 showed a better signal-to-background ratio than [^11^C]RO-643 and [^11^C]RO-963 in AD patients [98]. [^18^F]GTP1, a deuterated analogue of [^18^F]T808, was designed to improve the stability of radiotracer to defluorination. A clinical PET study of [^18^F]GTP1 successfully prevented tracer accumulation in the skull and clearly differentiated AD patients from healthy control subjects [98]. As mentioned above, ^18^F-labeled PBB derivatives, [^18^F]AM-PBB3 and [^18^F]PM-PBB3, have been developed by the National Institute of Radiological Sciences in Japan. These ^18^F-labeled PBB3 s showed a greater signal-to-background ratio and less off-target signals in the basal ganglia than [^11^C]PBB3. [^18^F]MK-6240, a novel pyridine isoquinoline amine derivative developed by Merck, showed a high sensitivity and specificity for PHF-tau binding [99]. In autoradiographic studies, MK-6240 showed high affinity to tangle-rich AD brain homogenates and large amounts of displaceable binding in the gray matter of AD brain sections [85]. Absence of off-target binding of MK-6240 to MAO-A and MAO-B was confirmed in preclinical studies as well [85]. Recent clinical studies have demonstrated that spatial patterns of MK-6240 binding were consistent with neuropathological staging of NFTs [100]. Unlike flortaucipir and THK5351, off-target binding of MK-6240 was not observed in the basal ganglia and choroid plexus although mild tracer retention was observed in the substantia nigra and meninges [100]. A novel tracer [^18^F]PI-2620 has also shown a lack of off-target binding in the basal ganglia [101]. Intriguingly, a preclinical analysis has suggested a high-affinity binding of PI-2620 to PSP and Pick’s disease brains [102]. To confirm these initial observations, several clinical studies are ongoing in non-AD patients.

## Conclusions and future prospects

Recent progress in the development of tau PET tracers has enabled the non-invasive monitoring of PHF-tau accumulation in aging and AD brains. This technology will further contribute to our understanding of the natural course of tau pathology progression and to the development of DMTs in AD. Nevertheless, the existing tau tracers have not been completely validated by autopsy studies as of yet, in contrast to amyloid PET tracers that have already been validated in clinical trials. To establish tau PET imaging as a biomarker of PHF-tau, imaging–autopsy correlation studies are necessary to confirm whether the amount and topographical distribution of the tracer retention truly reflect the formation of PHF-tau in the brain. It is also important to establish methods for accurate quantification of tracer binding, given that tau PET is used for therapeutic monitoring of PHF-tau density. There is as yet limited information about the speed and direction of tau pathology progression during the course of aging and AD. Current ongoing longitudinal studies may help clarify the natural history of PHF-tau formation.

The currently available tau tracers are less sensitive to detect non-AD tau lesions. Therefore, efforts should be focused on the development of PET tracers for selective imaging of 3R or 4R tauopathies. The same strategy could be applied to radiotracer development of other misfolded proteins such as α-synuclein and TDP-43. Neuroinflammation, which is characterized by the activation of glial cells, such as microglia and astrocytes, is considered to play a key role in the neurodegenerative process of various protein-misfolding diseases. Activated microglia stimulates the release of cytokines and chemokines and may cause synaptic dysfunction and neuronal loss. Reactive astrogliosis is also known to be strongly involved in AD progression and other neurodegenerative diseases. Sensitive PET tracers for activated microglia and reactive astrocytosis are indeed necessary to fully understand the neurodegenerative processes after misfolded protein accumulation. These tracers could be applied as surrogate markers of therapeutic efficacy of DMTs.
